# Trans RCED-UNet3+: a hybrid CNN-transformer model for precise lung nodule segmentation

**DOI:** 10.3389/fonc.2025.1654466

**Published:** 2025-10-16

**Authors:** Sadaf Raza, Razia Zia, Irfan Ahmed Usmani, Nouf Abdullah Almujally, Nada Alasbali, Muhammad Hanif

**Affiliations:** ^1^ Department of Electronic Engineering, Sir Syed University of Engineering & Technology, Karachi, Pakistan; ^2^ Department of Computer Science, Faculty of Engineering Science and Technology, Iqra University, Karachi, Pakistan; ^3^ Department of Biomedical Engineering, Salim Habib University (Formerly Barrett Hodgson University), Karachi, Pakistan; ^4^ Department of Information Systems, College of Computer and Information Sciences, Princess Nourah bint Abdulrahman University, Riyadh, Saudi Arabia; ^5^ Department of Informatics and Computer Systems, College of Computer Science, King Khalid University, Abha, Saudi Arabia; ^6^ Department of Informatics, School of Business, Örebro Universitet, Örebro, Sweden

**Keywords:** transformer bottleneck, lung nodule, hybrid loss function, RCED-UNet 3+, LIDC-IDRI

## Abstract

**Introduction:**

Precisely segmenting lung nodules in CT scans is essential for diagnosing lung cancer, though it is challenging due to the small size and intricate shapes of these nodules.

**Methods:**

This study presents Trans RCED-UNet3+, an enhanced version of the RCED-UNet3+ framework designed to address these challenges. The model features a transformer-based bottleneck that captures global context and long-range dependencies, along with residual connections that facilitate efficient feature flow and prevent gradient loss. To improve boundary accuracy, we employ a hybrid loss function that combines Dice loss with Binary Cross-Entropy, enhancing the clarity of nodule edges.

**Results:**

Evaluation on the LIDC-IDRI dataset demonstrates a notable advancement, as Trans RCED-UNet3+ achieves a Dice score of 0.990, exceeding the original model’s score of 0.984.

**Discussion:**

These findings underscore the value of merging convolutional and transformer architectures, delivering a robust approach for precise segmentation in medical imaging. This model enhances the detection of subtle and irregular structures, enabling more accurate lung cancer diagnoses in clinical environments.

## 1 Introduction

Lung cancer ranks among the deadliest and most frequently diagnosed cancers worldwide. Detecting it at an early stage is crucial, as timely intervention significantly improves survival chances. Often, its initial signs appear as small nodular formations in the lungs, making accurate identification and delineation essential for distinguishing between benign and malignant lesions (1). Computed Tomography (CT) scans remain the standard imaging technique for screening purposes, ability to produce detailed cross-sectional images of lung anatomy (2). However, the large number of CT scans generated in medical practice (3), along with the wide variation in nodule characteristics ranging in shape, size, surface texture, and internal composition, such as ground-glass opacity or calcifications, poses substantial difficulties for radiologists (4). Manually analyzing medical image slices by is not only a time-consuming but also subject to inconsistencies across different specialists. These challenges highlight the growing need for dependable automated segmentation methods, which can assist clinicians by facilitating faster analysis, enhanced consistency, and improved diagnostic accuracy.

Lung nodule segmentation methods can be grouped into three main categories: conventional machine learning, deep learning models, and more recent Transformer-based techniques. The earlier, conventional strategies rely heavily on manually defined characteristics like texture cues or shape-related details and tend to perform practically well when working with small datasets (5).Nonetheless, they often lack flexibility and struggle when applied to diverse or unseen data, partly due to their dependence on extensive preprocessing steps. Convolutional neural networks (CNNs), a core component of deep learning, have significantly advanced the field by recognizing diverse lung nodule characteristics (6). However, CNNs have a notable limitation: they concentrate mainly on nearby pixel patterns, which can limit their ability to capture the bigger picture or the spatial context that’s vital for precise segmentation. Transformer-based architectures aim to overcome this issue by using self-attention mechanisms, enabling them to account for distant spatial relationships within the image ultimately leading to more accurate results (7). However, these models typically demand large-scale datasets and considerable computing resources to achieve their best performance. To bridge this gap, new hybrid designs that fuse CNNs with Transformer elements are gaining traction, offering a practical mix of reliability and efficiency.

Transformer-driven architectures have made significant strides image processing, especially due to their capacity to model distant relationships in image data. Vision transformer ViT (8) is enhanced into CRViT to recognize images through random fourier features and inductive bias using causal relationships and convolutional down-sampling. The method has lower parameter sensitivity, and it performs better than baseline ViT and CNNs on small and medium datasets. DeiT (9) introduces a distillation technique to enable more efficient Transformer training. DETR (10) redefines object detection through set prediction using Transformer backbones. In the realm of medical imaging. Furthermore, TransUNet (11) has successfully integrated ViT into segmentation tasks, paving the way for further innovations in automated diagnostic tools. A number of hybrid CNN-Transformer-based models have been suggested to enhance lung nodule segmentation. SSLKD-UNet (12) is a semi-supervised teacher student approach. It utilizes coarse and fine annotations to assist in resolving the issue of data scarcity, though, further training makes it more complex. S3TU-Net (13) is a convolution system designed with a non-residual superPixel vision transformer and it scores high on both LIDC-IDRI and EPDB data sets. Nonetheless, it has a complicated multi-modular design. SW-UNet (14) uses a sliding-window attention to support global context modelling at computational efficiency. It generalizes to tumor datasets and is better at LUNA16. It does not expressly enhance feature propagation along the encoder-decoder path.

RCED-UNet3+ effectively overcomes various shortcomings of segmentation methods by incorporating dense connectivity and refined feature aggregation mechanisms (15). Although the model demonstrated enhanced performance, its convolutional structure remained limited in effectively capturing broader contextual dependencies, particularly when handling complex lung nodules such as small (<3mm) lesions or diffuse ground-glass opacities. To overcome these challenges, the Trans RCED-UNet3+ architecture was introduced, embedding Transformer modules within the RCED-UNet3+ framework to strengthen global feature representation.

The defining innovation of Trans RCED-UNet3+ is its hybrid design, which effectively merges the benefits of CNNs and transformers. Unlike TransUNet, which replaces the encoder with a transformer-based module (11), our approach embeds transformer-driven bottleneck connections within the encoder-decoder structure. This offers three major advantages: (1) it retains the CNN’s strength in local feature extraction, (2) enhances global contextual understanding through well-placed self-attention mechanisms, and (3) optimizes computational efficiency by restricting transformer operations to bottleneck layers. Additionally, the model employs residual connections to improve gradient propagation and mitigate information loss in deeper layers. This design fosters superior feature fusion and enhances the detection of small nodules. By addressing existing gaps, the proposed model aims to improve segmentation accuracy and reliability, making it more aligned with clinical requirements.

This study contributes the following:

1. This work enhances the RCED-UNet3+ architecture by incorporating transformer-based bottlenecks and residual connections, resulting in the Trans RCED-UNet3+ model. The proposed model improves segmentation precision and reliability by combining the convolutional neural networks’ capacity for extracting detailed local features with the transformer architecture’s strength in modeling global context and long-range relationships.
2. The study thoroughly explores how transformers contribute to medical image segmentation, particularly in detecting lung nodules. Robust experiments have been conducted to evaluate how effectively they capture long-distance dependencies, enhance feature quality, and mitigate performance degradation within the network. The impact of transformer integration is assessed in terms of segmentation accuracy, adaptability across different nodule sizes, and robustness to variations in medical imaging datasets.
3. To overcome the issue of limited data in transformer-based models, we implemented data augmentation as a fundamental approach. Since transformers require extensive datasets to effectively learn spatial relationships, augmentation was applied to synthetically enlarge the dataset and strengthen the model’s ability to generalize.
4. To demonstrate the effectiveness of Trans-RCEDUNet3+, an extensive evaluation is conducted against leading segmentation approaches. Various benchmark datasets are used to measure performance based on Dice Score and IoU. The findings highlight notable improvements in detecting small and complex lung nodules, confirming the benefits of integrating transformers into RCED-UNet3+.

## 2 Literature review

Medical image segmentation has undergone substantial advancement, progressing from traditional methods to advanced frameworks driven by deep learning techniques. Early segmentation strategies included thresholding, region-growing (16), edge detection, iterative local thresholding, active contour models, and clustering algorithms (17). Although these methods required significant manual effort for parameter tuning and preprocessing. Consequently, they often lacked the precision and flexibility necessary to manage the intricate anatomical details observed in medical scans.

Deep learning techniques have increasingly demonstrated superior performance in medical image analysis, offering improved accuracy, robustness, automated feature extraction, and greater generalization across various imaging scenarios. The fundamental tools for semantic segmentation is 2D segmentation networks, which is structures with an encoder to capture feature extraction and decoder to reconstruct the pixel wise classification maps (18). Long et al. (19) introduces Fully Conventional Network (FCN) which enables direct pixel-level prediction in an end-to-end learning framework. Ronneberger et al. (20) proposed U-Net architecture by integrating skip connections between corresponding encoder and decoder layers to address the loss of spatial resolution. While the U-Net architecture achieved considerable success, it faced limitations in accurately segmenting very small or indistinct structures, which led to the development of more advanced variants. To address these challenges, Huang et al. in 2020 (21) proposed UNet3+, which integrates full-scale skip connections and multi-scale feature fusion to improve spatial precision. Subsequently, Zhou et al. (22) extended this idea through UNet++, introducing densely nested skip connections aimed at minimizing the semantic gap between encoder and decoder representations. Building upon these improvements, Xiao et al. (23) presented SAUNet++, combining Squeeze-and-Excitation Residual (SER) modules with Atrous Spatial Pyramid Pooling (ASPP) to suppress irrelevant background noise and enable rich multi-scale feature extraction. More recently, Yao et al. (24) further refined the design by incorporating attention mechanisms and residual blocks, thereby enhancing deeper feature learning and achieving better segmentation of fine-grained structures. Wu et al. (25) brings forward a set of architectural improvements for segmentation tasks. The conventional U-Net encoder is substituted with a residual framework inspired by ResNet, followed by the integration of an atrous spatial pyramid pooling (ASPP) unit to capture multi-scale context. Additionally, the decoder is refined by incorporating a cross-fusion module that leverages both spatial and channel-level attention mechanisms to enhance feature representation.

In 2021, Wang et al. (26) introduced an improved U-Net variant by embedding Squeeze-and-Excitation (SE) blocks (27) at each decoder stage. These SE modules calculated attention weights across feature channels, amplifying the most informative signals and significantly improving the segmentation of small areas. Although convolutional neural networks excel in various image analysis applications, they often struggle to effectively capture long-range interactions and manage complex, high-dimensional feature representations (11).

Recent years have seen the emergence of Transformer-based architectures (28). Vision transformer-based models have been found to provide better lung segmentation of CXR images by effectively extracting both global and local features that allow it to outperform U-Net in accuracy and robustness (29). This has led to hybrid models that combine convolutional network local feature extraction with Transformer global contextual understanding. In 2021, Chen et al. (11) proposed TransUNet, a hybrid architecture that embeds Transformer modules within the U-Net framework. Specifically, it integrates multiple Transformer blocks between the encoder and decoder stages. Nonetheless, naively stacking several Transformer layers post-encoder often results in overfitting, which is especially detrimental when working with limited datasets. To overcome this limitation, TransFuse (30) presents a dual-path architecture that operates in parallel one branch based on CNNs and the other on Transformers combining their respective features via a dedicated fusion mechanism. Chen et al. (31) conducted a comprehensive comparison of eight state-of-the-art neural network models UNet, SegNet, GCN, FCN, DeepLabV3+, PSPNet, TransUNet, and SwinUNet for lung nodule segmentation in CT scans, using the LIDC-IDRI dataset. The study also examined the influence of preprocessing techniques, including region-of-interest (ROI) cropping and lung contour overlays, on segmentation accuracy. Among the tested models, Transformer-based architectures, particularly TransUNet, achieved the highest performance, recording a Dice score of 0.871 across four experimental settings and outperforming conventional CNN-based approaches. UCTransNet (32) addresses semantic inconsistencies between encoder and decoder by substituting conventional skip connections with Transformer-based modules that leverage channel-wise self-attention. UTNet (33) retains the encoder-decoder structure of the U-shaped architecture but inserts self-attention layers after each module pair; however, the proliferation of these attention layers can degrade performance on small-scale datasets. ResT (34) introduces a multi-scale Transformer design that enhances inter-head communication among self-attention modules using lightweight convolutional blocks. MCTrans (35) combines both self-attention and cross-attention techniques to model semantic correlations and enrich feature maps, enabling effective multi-scale dependency capture across different feature levels.

**Table 1** presents a comparative analysis of these contributions, outlining the strengths and limitations of each approach and demonstrating the progression of segmentation models over time. This review underscores the necessity for continued research to refine segmentation frameworks, ensuring an optimal balance between computational efficiency, accuracy, and reliability for real-world clinical applications.

**Table 1:** Literature summary of medical image segmentation.

Reference	Year	Study Domain	Dataset	Model	Description	Limitations
Dehmeshki et al. (16)	2008	Lung nodule segmentation	Thoracic CT scans	Region growing approach	Proposes a region-growing technique for segmenting pulmonary nodules by integrating fuzzy connectivity with contrast-based methods to enhance accuracy. It determines the optimal seed point and applies sphericity constraints for precise boundary identification.	The method has certain limitations, including dependence on a seed point, difficulties in handling highly intricate nodules, and processing time limitations.
El-Regaily et al. (17)	2017	Lung nodule detection	CT scans	Thresholding and Region growing approach	This study introduces a fully automated CAD system for lung nodule detection , utilizing a multi-stage process with 3D Region Growing and morphological operations.	It struggles with highly transparent ground glass nodules and has a high false positive rate.
Long et al. (19)	2015	Semantic segmentation	PASCAL VOC	Fully Convolutional Network (FCN)	Introduced FCNs for end-to-end pixel-level image segmentation. FCNs replace fully connected layers with convolutional layers, enabling segmentation of images of any size.	Limited global context modeling; struggles with small or occluded targets.
Ronneberger et al. (20)	2015	Biomedical image segmentation	ISBI cell tracking challenge	U-Net	A U-Net architecture was introduced, featuring a symmetrical encoder-decoder structure integrated with skip connections, specifically designed for segmenting biomedical images U-Net is highly effective but struggles with small or occluded targets.	Exhibits difficulty in accurately detecting small or partially obscured objects.
Huang et al. (21)	2020	Medical image segmentation	Synapse multi-organ CT dataset	UNet3+	Introduced UNet3+, which leverages multiscale features and full-scale skip connections to improve segmentation accuracy and efficiency.	Computationally expensive; requires large datasets for training.
Zhou et al. (22)	2018	Medical image segmentation	Liver tumor segmentation (LiTS)	UNet++	Proposed UNet++, which enhances U-Net by incorporating dense and nested skip connections to bridge the semantic gap between encoder and decoder.	Increased complexity; may overfit on small datasets.
Xiao et al. (23)	2023	COVID-19 lesion segmentation	COVID-19 CT slices	SAUNet++	Developed SAUNet++, which integrates SER and ASPP modules into UNet++ to improve segmentation of COVID-19 lesions from CT slices.	Limited to COVID-19 lesions; may not generalize well to other medical imaging tasks.
Yao et al. (24)	2020	Scleral blood vessel segmentation	Scleral blood vessel dataset	CLAW U-NET	Introduced CLAW U-Net, a U-Net-based architecture that integrates residual blocks and attention mechanisms to enhance the segmentation of scleral blood vessels.	Specialized for scleral blood vessels; may not generalize to other medical imaging tasks.
Wang et al. (26)	2021	Liver segmentation in CT	LiTS17 and SLiver07.	SAR-U-Net	Proposed SAR-U-Net, an enhanced residual U-Net architecture that integrates Squeeze-and-Excitation (SE) modules and atrous spatial pyramid pooling (ASPP) to enable automated liver segmentation in CT images.	Limited to liver segmentation; computationally intensive.
Chen et al. (11)	2021	Medical image segmentation	Synapse multi-organ CT dataset	TransUNet	Proposed TransUNet, which integrates Transformer structures into U-Net networks to capture global information and improve segmentation accuracy.	Prone to overfitting; requires large datasets; computationally expensive.
Zhang et al. (30)	2021	Medical image segmentation	( CVC-Clinic DB) (ISIC2017)	TransFuse	Introduced TransFuse, a dual-branch architecture that combines CNN and Transformer features through a fusion module for medical image segmentation.	Limited feature interaction during training; computationally expensive.
Wang et al. (32)	2022	Medical image segmentation	Synapse multi-organ CT dataset	UCTransNet	Proposed UCTransNet, which replaces traditional skip connections in U-Net with a Transformer-based channel-wise attention mechanism to reduce semantic discrepancies between encoder and decoder.	Focuses on channel-wise attention; may not fully exploit global feature information.
Gao et al. (33)	2021	Medical image segmentation	Siemens; Philips, GE, Canon	UTNet	Developed UTNet, a hybrid Transformer architecture that places self-attention layers after each encoder-decoder module for medical image segmentation.	Excessive self-attention layers may hinder performance on small datasets.
Zhang and Yang (34)	2021	Visual recognition	ImageNet	ResT	Introduced ResT, a multi-scale Transformer that enhances information exchange between different self-attention heads using a simple convolutional block.	Limited to visual recognition tasks; may not generalize to medical image segmentation.

Although remarkable steps have been made in medical image segmentation, a number of difficulties still remain. These include reliably detecting very small, managing variability across different datasets, and addressing the tendency of complex models to overfit. Overcoming such issues requires ongoing refinement, particularly through the development of more effective hybrid modeling strategies. In this study, we aim to contribute to that effort by investigating new ways to combine architectural components in order to enhance segmentation outcomes. Our focus lies in improving accuracy particularly in identifying small lung nodules within CT scans. To this end, we introduce a architecture that merges the RCED-UNet3+ framework with Transformer-based elements. This combination is intended to better capture wide-ranging contextual information while minimizing performance degradation across layers.

## 3 Materials and methods

**Figure 1:**
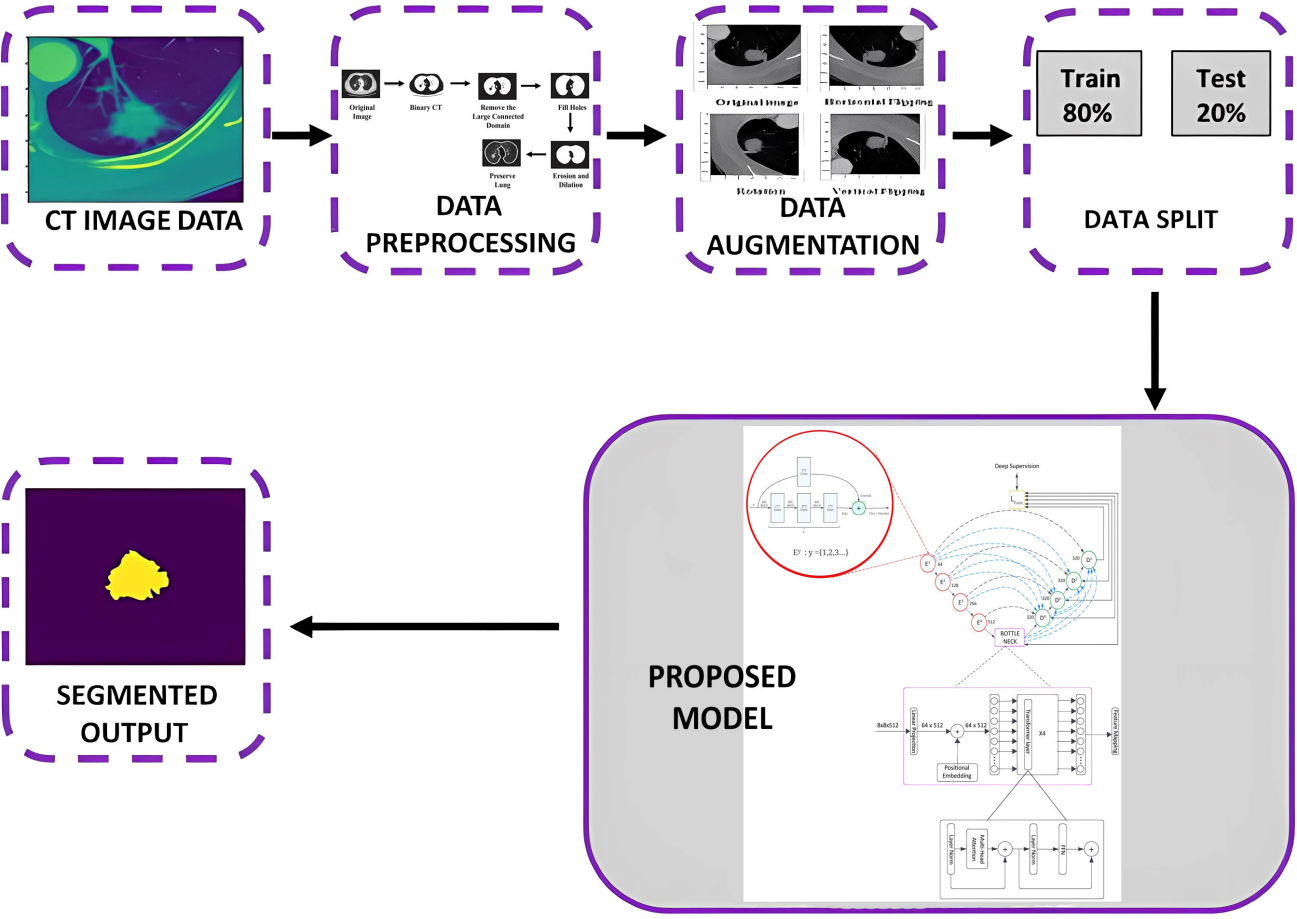
Overview of the proposed Trans RCED-UNet3+ architecture for lung nodule segmentation.

As discussed in the introduction, we have adopted the RCED-UNet3+ framework and further extended it by integrating a Transformer component, resulting in the Trans RCED-UNet3+ architecture, as depicted in **Figure 1**. This improved design maintains the core strength of residual connections, which support the encoder in extracting high-level features. At the same time, it introduces a Transformer-based bottleneck layer designed to recognize global spatial patterns and interpret contextual information throughout the image. While residual pathways play a key role in preventing performance degradation during deep learning, the Transformer component enhances the model’s ability to generalize when faced with unfamiliar data. To further support accurate segmentation, a hybrid loss function has been incorporated, aimed at minimizing pixel-level classification errors and improving the distinction between foreground and background regions. The following sections delve into the architectural improvements and technical implementation details of these modifications.

### 3.1 Dataset

This study utilizes LIDC-IDRI, a database of lung CT scans which frequently used in medical imaging studies. This dataset comprises 1,018 CT scans collected from 1,010 unique patients, each of which was annotated by four certified radiologists. It provides chest CT images stored in DICOM format, accompanied by XML files. All scans are standardized to a resolution of 512 × 512 pixels, formatted in three channels, and normalized using Hounsfield Units (HU) to ensure consistency in intensity values. The LIDC-IDRI dataset is used for developing and testing deep learning models due to its comprehensive annotations and uniform imaging standards designed for lung nodule segmentation and the early diagnosis of lung cancer.

### 3.2 Preprocessing

**Figure 2:**
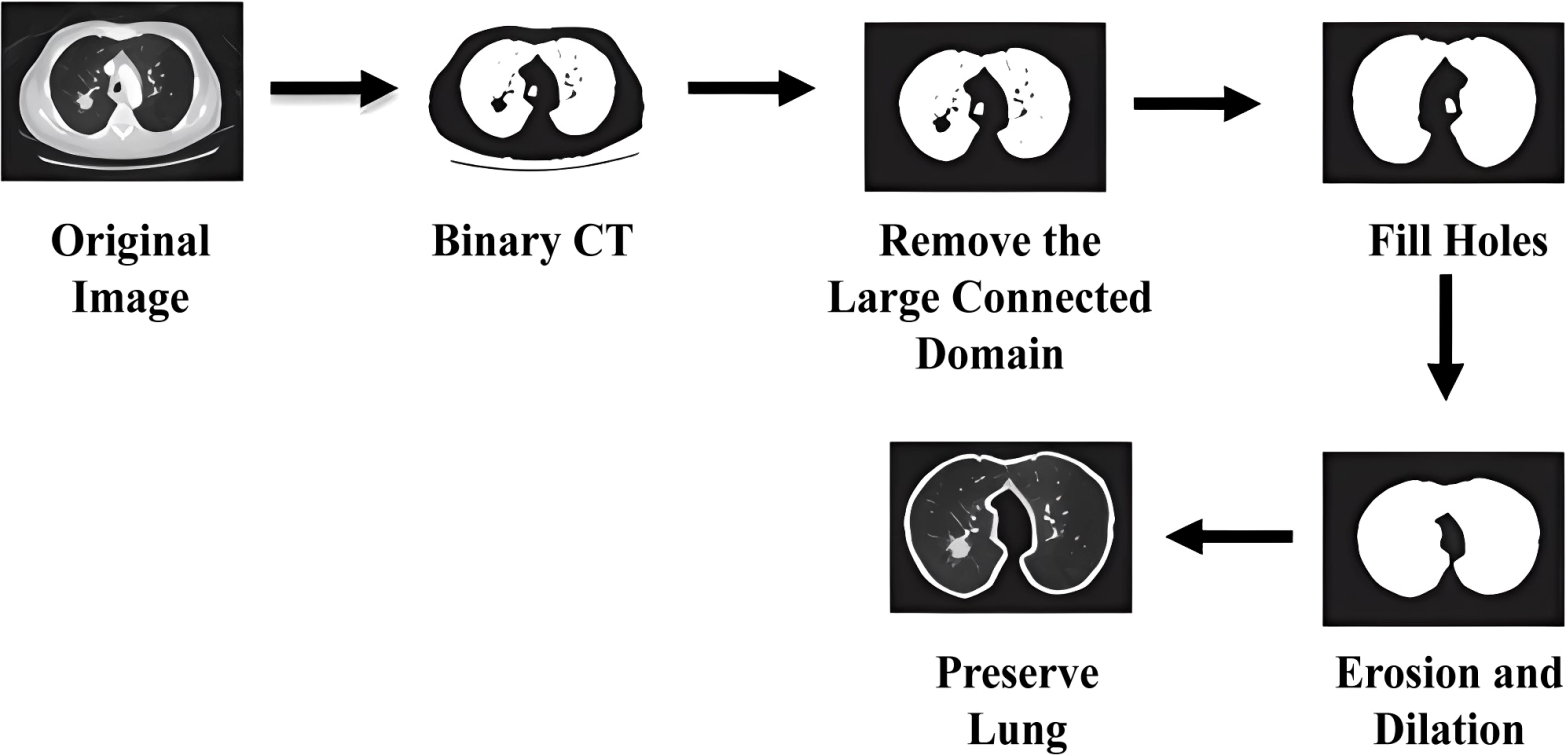
Methodological overview of lung region segmentation in CT imaging.

The preprocessing stage begins by isolating the lung parenchyma from each CT scan, ensuring that unrelated anatomical structures such as bones and soft tissue are excluded. Because lung nodules are embedded within this parenchymal tissue, accurately identifying and extracting this region is critical for improving segmentation accuracy and reducing the likelihood of false positives. In CT images, the parenchyma appears as a darker, low-density zone, distinctly contrasted by the lighter shades representing surrounding musculature. The segmentation process initiates with a thresholding technique that converts the grayscale scan into a binary image displaying the lung regions in black and the rest of the anatomy in white. To address interruptions caused by dense regions within the lungs, morphological dilation is subsequently applied. This step broadens the segmented lung areas and bridges any disjointed sections, ultimately creating a more complete and continuous mask of the lung parenchyma. The overview of lung region segmentation in CT imaging is shown in **Figure 2** (15).

### 3.3 Data augmentation

**Figure 3:**
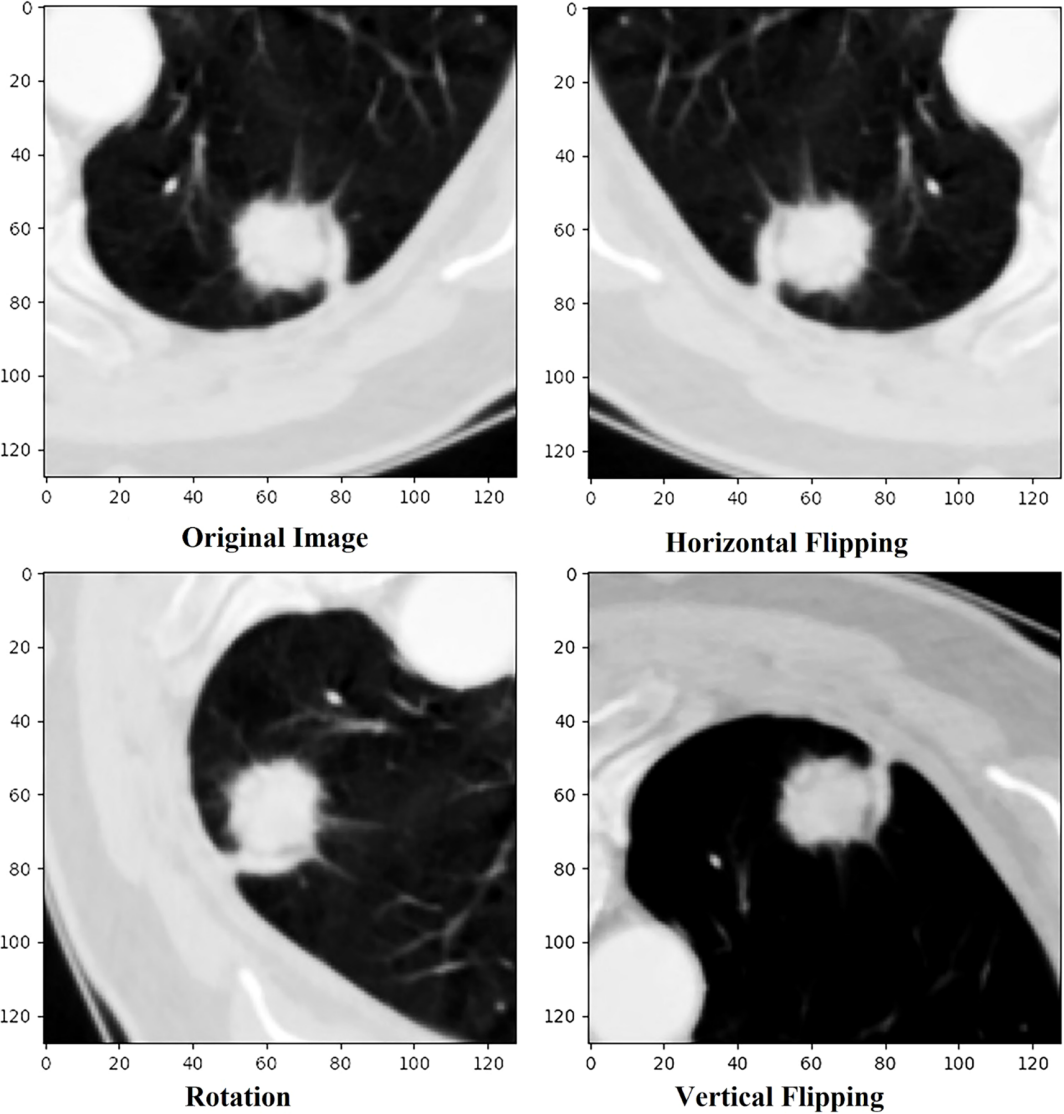
Original CT slice with three variants of data augmentation techniques.

To overcome the limitation of small training dataset inherent in transformer-based segmentation models, data augmentation serves as a critical preprocessing step. Transformers demand substantial datasets to effectively learn complex spatial hierarchies; therefore, augmentation plays a key role in synthetically increasing dataset diversity and enhancing model generalization. This study employs three augmentation techniques horizontal flip, vertical flip, and rotation resulting in a fourfold expansion of each input sample and generating a total of 62,200 augmented instances. These transformations introduce spatial perturbations that enhance the model perform well on data not included in training while minimizing the chances of over fitting. Experimental evidence from prior research supports the efficiency of such approaches; for example, Ahmed et al. (36) present an ecologically valid augmentation method by integrating real pathological stroke lesions into healthy brain MRI scans, demonstrating notable improvements in segmentation accuracy for transformer-based networks. Shah et al. (37) highlighted that various data augmentation techniques have the potential to substantially enhance the resilience and functionality of Vision Transformer models in medical image segmentation to adversarial risk and scant data challenges, among others. These studies highlight the effectiveness of augmentation techniques in optimizing transformer-based segmentation models, aligning with the approach used in this work. The augmentation methods, horizontal flipping, vertical flipping, and rotation are implemented to achieve geometric transformation invariance, as depicted in **Figure 3** (15).

### 3.4 Proposed methodology

**Figure 4:**
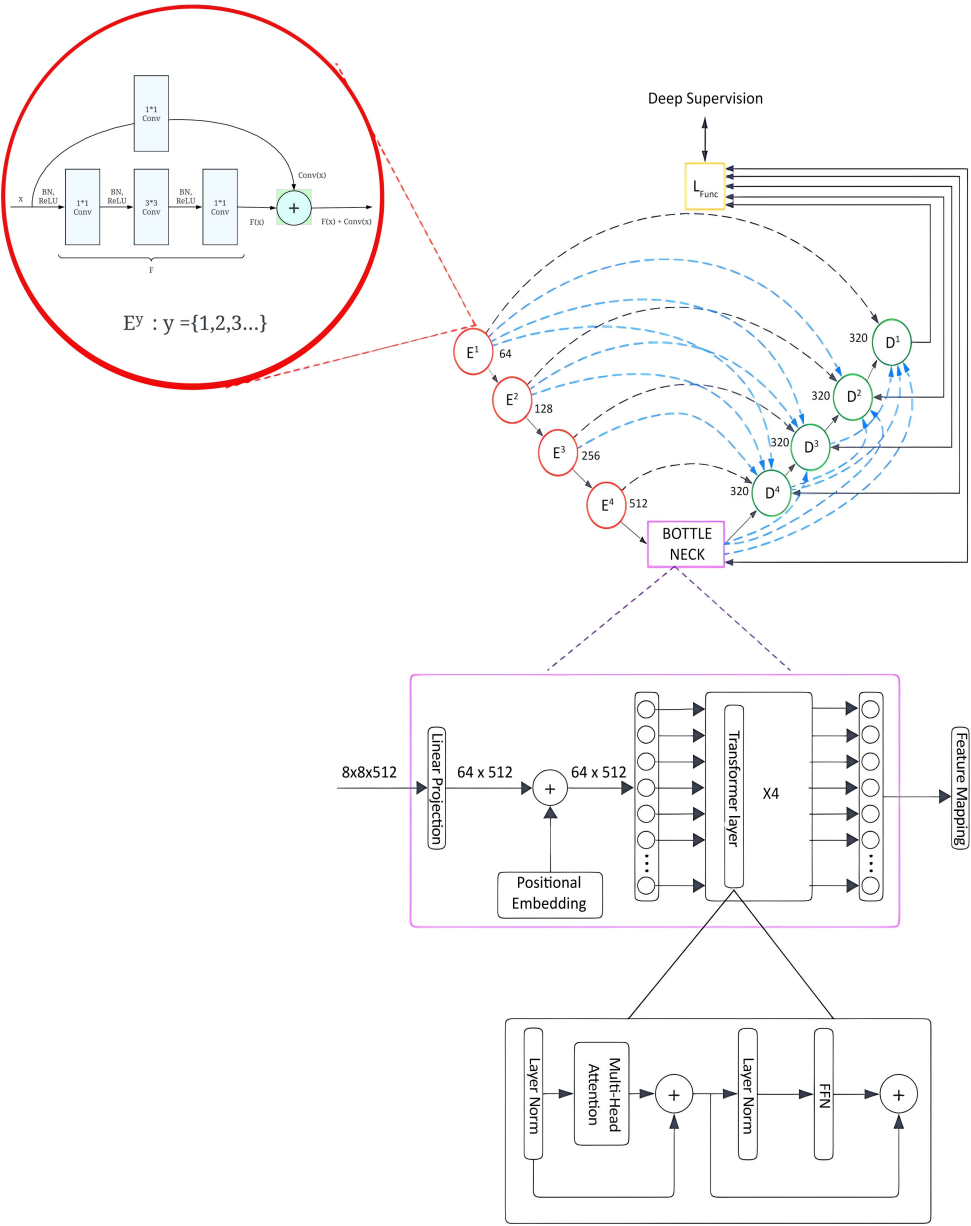
Trans RCED-UNet3+ architecture with a transformer bottleneck for global context modeling, integrating residual dense blocks, deep supervision, and multi-head self-attention.

This section presents the architectural advancements introduced in the Trans RCED-UNet3+ model, an evolution of the previously established RCED-UNet3+ (15). The proposed model integrates a bottleneck transformer module aimed at enhancing segmentation performance by effectively modeling long range spatial dependencies and global contextual information, while preserving the strength of the original model’s residual connectivity. The underlying framework of Trans RCED-UNet3+ builds upon the RCED-UNet3+ structure, which is characterized by the strategic incorporation of residual networks within both the encoder and decoder pathways. These residual connections facilitate efficient gradient flow, address vanishing gradient challenges. **Figure 4** presents the proposed model architecture, which facilitates effective feature flow across the entire network while employing multi-head self-attention to capture global contextual relationships.

#### 3.4.1 Residual connection networks

The RCED-UNet3+ architecture leverages residual connections to facilitate stable and efficient information flow across network layers, addressing the degradation problem commonly observed in deep convolutional architectures. These residual pathways ensure uninterrupted gradient propagation during both forward and backward passes, thereby enhancing training stability and convergence. Each residual block consists of convolutional structure employing kernel sizes of 1 * 1, 3 * 3, and 1 * 1, respectively, which collectively optimize local and cross-channel feature extraction. To ensure stable training and improve regularization, batch normalization is performed before convolution, followed by the application of a ReLU activation function. This integration standardizes intermediate outputs, minimizes internal covariate shifts, and reduces overfitting throughout the training process. The residual connection can be mathematically formulated as shown in Equation (1).


(1) RC = x + F(x)

where x  denotes the input feature and F(x)  represents the non-linear transformation learned by the convolutional subnetwork. To ensure element-wise addition, a 1 * 1 convolutional projection with zero-padding is applied to x , the residual computation is therefore defined in Equation (2).


(2) RC = Conv(x) + F(x)

Structural consistency within the residual block is achieved by aligning both spatial and channel dimensions. By incorporating residual connections, the model improves feature representation, which enables gradients to flow more effectively during training. Moreover, they reduce the loss of features in deeper layers, ultimately leading to higher segmentation accuracy.

Transformer-based bottleneck is added in the Trans RCED-UNet3+ to improve its ability to grasp long-range spatial dependencies and global contextual information. Conventional convolutional layers, in contrast, are limited to localized feature extraction and are unable to capture global contextual information. By integrating these global contextual representations with detailed local information, the architecture can achieve more robust and precise segmentation results.

#### 3.4.2 Transformer-driven bottleneck

The integration of a transformer-based bottleneck into the Trans RCED-UNet3+ architecture improves the segmentation accuracy, which recognizes long-range spatial dependencies and global contextual information in lung CT images. Conventional convolutional neural networks (CNNs) are limited to investigating local regions, but the transformer module overcomes this by using self-attention throughout the image. This component is based on Vision Transformer (ViT), where feature maps from the encoder are segmented into uniform patches, which are converted into embeddings and processed through multi-head self-attention (MHSA). This process enables the network to model complex, non-local dependencies and enhance its understanding of spatial relationships across the medical scan. The fundamental components of the architecture include:

##### 3.4.2.1 Patch tokenization

O Feature maps from the encoder are divided into smaller patches and transformed into sequential 1D tokens.
O The segmented patches are transformed into a higher-dimensional representation through a linear projection layer.

##### 3.4.2.2 Spatial encoding

O Since transformers do not inherently capture spatial structures, positional encoding is added to retain location-based information.
O This ensures the model can distinguish between different regions within the image.

##### 3.4.2.3 Multi-head self-attention

O The multi-head self-attention mechanism enables the model to focus on multiple regions of the image simultaneously.
O Each attention head generates a feature representation by computing a weighted combination of the input features, as defined in Equation (3).

(3) $$Attention\;(Q,\;K,\;V)=softmax\;(\frac{QK^{T}}{\sqrt{d_{k}}})\;V$$

Here, Q, K, and V represent the query, key, and value matrices derived from input embedding.

This mechanism allows the model to highlight relevant lung regions while reducing interference from background noise.

##### 3.4.2.4 Feed-forward network & residual links

o After self-attention, the extracted representations are passed through a fully connected feed-forward module (FFN).
O Residual connections are included to stabilize gradient flow and improve training efficiency.

The output from the transformer is up sampled and processed through convolutional layers to produce refined feature maps that incorporate improved global contextual information.

#### 3.4.3 Comparison with traditional CNN-based methods

##### 3.4.3.1 Improved global feature capture

O CNNs are primarily designed to extract localized spatial details, which may limit their ability to detect relationships between distant lung nodules.
O Transformers employ self-attention to capture global relationships, which contributes to more precise and reliable segmentation outcomes.

##### 3.4.3.2 Computational complexity

O The processing demand of the self-attention component in transformer architectures grows quadratically with sequence length *O*(*n* ^2^), making it more resource-intensive than CNNs, which operate at O(n). However, by incorporating a transformer bottleneck instead of a full transformer framework, a balance between computational efficiency and segmentation performance is achieved.

## 4 Experimental setup

The proposed model was trained and tested using the LIDC-IDRI dataset, a reputable and extensively used resource in lung nodule segmentation research. Preprocessing steps included isolating the lung parenchyma through binarization and morphological operations. Since transformers require large datasets to learn spatial dependencies effectively, augmentation helps artificially increase the dataset size and improve model generalization, augmentation techniques such as vertical and horizontal flipping, as well as rotation, were applied.

### 4.1 Training configuration

The model was trained using the Stochastic Gradient Descent (SGD) optimizer, configured with a learning rate of 1e-3, a dropout rate of 0.15, and a batch size of 32. To improve segmentation accuracy, the hybrid loss function from the RCED-UNet3+ model was retained, which penalizes inaccuracies in both foreground and background classification. This hybrid loss function combines dice Loss and binary cross-entropy Loss. Dice loss is particularly effective for assessing the overlap between predicted segmentation and ground truth, while BCE loss penalizes incorrect classifications.

Let C denotes the ground truth mask, S the predicted segmentation. The dice loss is defined in Equation (4)


(4) $$l_{DLS\;=}\;1-\;\frac{2CS+1}{C+S+1}$$

The binary-cross entropy loss is denoted as $l_{BCE}$ and is expressed in Equation (5):


(5) $$l_{BCE=}\;Clog(s)\;+\;[(1-Clog(1-S)]$$

The hybrid loss function $l_{H}$ which combines both Dice and BCE losses, is expressed in Equation (6).


(6) $$l_{H\;\;}=\;l_{DLS}+\;l_{BCE}$$

### 4.2 Transformer-based enhancements

The Trans-RCEDUNet3+ model introduces a transformer-based bottleneck to improve segmentation accuracy. One enhancement explored in this study is an attention-weighted loss function, where Dice and BCE losses are adjusted using an attention score derived from the transformer module. This prioritization aids in refining segmentation, particularly for small and complex lung nodules. The attention-weighted Dice loss function is given in Equation (7)


(7) $$L_{Attn_{\_Dice}}=1-\;\frac{2\;\Sigma \;Ai\;Pi\;Gi}{\Sigma \;Ai\;Pi+\;\Sigma \;Ai\;Gi+\;є}$$

Where:

*A_i_* represents the attention weight assigned to each pixel
*P_i_* and *G_i_* are the predicted and ground truth values respectively.
*Є* is small constant to prevent division by zero.

### 4.3 Hyper parameter selection and optimization

To ensure optimal performance of Trans RCED-UNet3+, a systematic hyperparameter tuning process was conducted, focusing on parameters critical for both CNN-based feature extraction and transformer-based self-attention mechanisms.

#### 4.3.1 Tuning of optimizer and learning rate parameters

O The learning rate was tuned using a grid search over the range {1e-4, 5e-4, 1e-3, 5e-3, 1e-2} with 1e-3 providing the best balance between convergence speed and stability.
O The Stochastic Gradient Descent (SGD) optimizer with momentum (0.9) and weight decay (1e-4) was used.
O AdamW was also tested, showing similar performance but with slightly higher memory usage, making SGD the preferred choice.

#### 4.3.2 Transformer-specific hyperparameter tuning

O Number of Attention Heads: We tested {4, 8, 12} attention heads, with 8 heads yielding the best trade-off between accuracy and computational efficiency.
O Feed-Forward Network (FFN) Dimensionality: Evaluated {512, 1024, 2048}, with 1024 providing optimal performance.
O Dropout Rate: A dropout rate of 0.15 was retained, as it offered a good balance between generalization and overfitting prevention. Lower dropout values (0.05, 0.1) led to overfitting, while higher dropout values (0.2, 0.3) negatively impacted convergence.

#### 4.3.3 Batch size and training stability

O To accommodate the memory constraints of the NVIDIA RTX 3090 GPU, a batch size of 32 was selected.
O Increasing the batch size to 64 led to unstable training behavior, attributed to greater variability in gradient updates.

#### 4.3.4 Grid search vs. random search for optimization

O A grid search was initially conducted for learning rate, optimizer, and dropout rate.
O A random search was used for transformer-specific parameters (attention heads, FFN size) due to the high-dimensional search space (**Table 2**).

**Table 2:** Hyperparameter configuration for trans RCED-UNet3+:.

Hyperparameter	Value	Justification
Learning Rate	1e-3	Balanced stability and convergence
Optimizer	SGD (momentum 0.9)	More stable than AdamW for this architecture
Dropout Rate	0.15	Prevents overfitting without affecting training speed
Batch Size	32	Optimal for GPU memory and stable training
Number of Attention Heads	8	Best trade-off between accuracy and computation
FFN Dimensionality	1024	Improved segmentation performance
Training Epochs	50	Convergence achieved within this range

### 4.4 Evaluation metrics

To further investigate the model’s performance, we calculated Accuracy, Precision, and Recall using standard classification outcomes: true positives (TP), true negatives (TN), false positives (FP), and false negatives (FN).

• Accuracy, which measures the proportion of correctly classified pixels relative to the total pixel count in the segmentation output, is defined in Equation (8)


(8) $$Accuracy=\frac{TP+TN}{TP+FP+TN+FN}$$

• Precision, which reflects the proportion of lung nodule pixels accurately identified by the model out of all pixels classified as nodules, is defined in Equation (9).


(9) $$Precision=\frac{TP}{TP+FP}$$

• Recall, which calculates the fraction of pixels correctly recognized as belonging to a lung nodule compared to the total number of lung nodule pixels, is defined in Equation (10).


(10) $$Recall=\;\frac{TP}{TP+FN}$$

These metrics, along with the Intersection over Union (IoU) and the Dice coefficient, provided a comprehensive assessment of the model’s segmentation accuracy and effectiveness.

## 5 Results and discussion

The performance of the Trans-RCEDUNet3+ model is evaluated using segmentation metrics: the Dice Similarity Coefficient (DSC) and Intersection over Union (IoU). These metrics were selected for their ability to examine how accurately the model segments lung nodules within CT scan images.

### 5.1 Ablation study

The addition of residual connections and transformer modules to UNet3+ architecture systematically over each of the proposed building blocks provided a more detailed analysis of the contribution of each element to the proposed framework, realized through an ablation study. The analysis shows the contribution of individual modules and explains how they contributed to the further development of the segmentation of the lung nodules. **Table 3** presents the results of four different versions, namely (i) UNet3+ (baseline), (ii) RCED-UNet3+ with residual connections between encoder and decoder, (iii) TransUNet3+ with a transformer bottleneck, and (iv) Trans-RCEDUNet3+. The results show that the residual connections added to UNet3+ regularize the training procedure and solve the vanishing gradient problem to get improved Dice and IoU scores. Even within the context of the transformer bottleneck in UNet3+, the model can capture long-range relationships and global contextual information, which further improves the quality of the segmentation. Finally, the combination of two residual connections and transformer modules in Trans-RCEDUNet3+ provides the best overall performance.

**Table 3:** Quantitative Assessment of Segmentation Accuracy for Baseline UNet3+, RCED-UNet3+, Trans UNet3+ and Trans RCED-UNet3+.

Model	Mean IoU	Mean dice
UNet3+	0.932	0.965
RCED-UNet3+	0.979	0.984
Trans UNet3+	0.978	0.984
Trans RCED-Net3+	**0.981**	**0.990**

Bold values correspond to the proposed model.

### 5.2 Comparative analysis

The comparative analysis is based on the ablation study and offers a comprehensive assessment of the four model variants, namely UNet3+, RCED-UNet3+, Trans-UNet3+, and Trans-RCEDUNet3+. **Table 3** shows a quantitative evaluation and **Table 4** shows the computational performance and efficiency of these models. Based on the findings, Trans RCED-UNet3+ has superior performance in segmentation than baseline models. Despite the practicality of the performance improvement brought by utilizing residual connections in combination with transformer modules, the complexity of the model prevents its application in resource-limited settings as the hyperparameters need to be carefully fine-tuned and the training process takes more time. It was also evaluated using a single dataset, other studies will be conducted in future to evaluate performance on diverse datasets using different scanners, institutions and imaging protocols to ascertain additional generalizability and clinical applicability.

**Table 4:** Computational analysis and performance metrics for baseline UNet3+, RCED-UNet3+ model, Trans UNet3+, and Trans RCED-UNet3+.

Model	Params (M)	FLOPs (GMac)	Epoch Time (s)	Epoch time (h)	Total time (50 epoch, h)	Inference (ms/img, B = 1)	Peak GPU memory (GB)
UNet3+	24.09	641.45	3 463.7	0.962	48.11	65	13.3
RCED-UNet3+	26.99	793.83	4 286.6	1.191	59.54	80	14.5
TransUNet3+	29.50	880.10	4 754.9	1.321	66.04	90	15.2
Trans RCED-Net3+	31.27	924.28	4 991.0	1.386	69.32	94	16.0

To support these findings, pair wise t-tests were conducted on values of Dice Score between the four models. The t -test in **Table 5** indicates that most of the model comparison provided statistically significant differences (p < 0.001), and this justified the performance improvement strength in architecture. Specifically, a significant improvement over the baseline UNet3+ was observed in RCED-UNet3 +, Trans UNet3+ and Trans RCED-UNet3 +. Also, Trans RCED-UNet3+ has continuously performed better than RCED-UNet3+ and Trans UNet3+, indicating its superiority in segmentation. The comparison that was not statistically significant was that between RCED-UNet3+ and Trans UNet3+ (p = 0.43); consequently, both shows indicated the similarity in performances. Overall, these findings verify that integration of residual connections and transformer modules into the UNet3+ architecture leads to large performance gains and that Trans RCED-UNet3+ offers the most consistent and informative advancement.

**Table 5:** Statistical significance t-test of dice scores between UNet3+, RCED-UNet3+, Trans UNet3+, and Trans RCED-UNet3+ models.

Comparison	t-value	p-value	Significance
UNet3+ vs RCED-UNet3+	-33.4642	1.85 × 10^−153^	Significant
UNet3+ vs Trans UNet3+	-28.8585	9.06 × 10^−136^	Significant
UNet3+ vs Trans RCED-UNet3+	-44.8444	3.42 × 10^−219^	Significant
RCED-UNet3+ vs Trans UNet3+	0.7871	0.4314	Not Significant
RCED-UNet3+ vs Trans RCED-UNet3+	-20.4716	8.50 × 10^−80^	Significant
Trans UNet3+ vs Trans RCED-UNet3+	-15.4285	4.81 × 10^−48^	Significant

**Figure 5:**
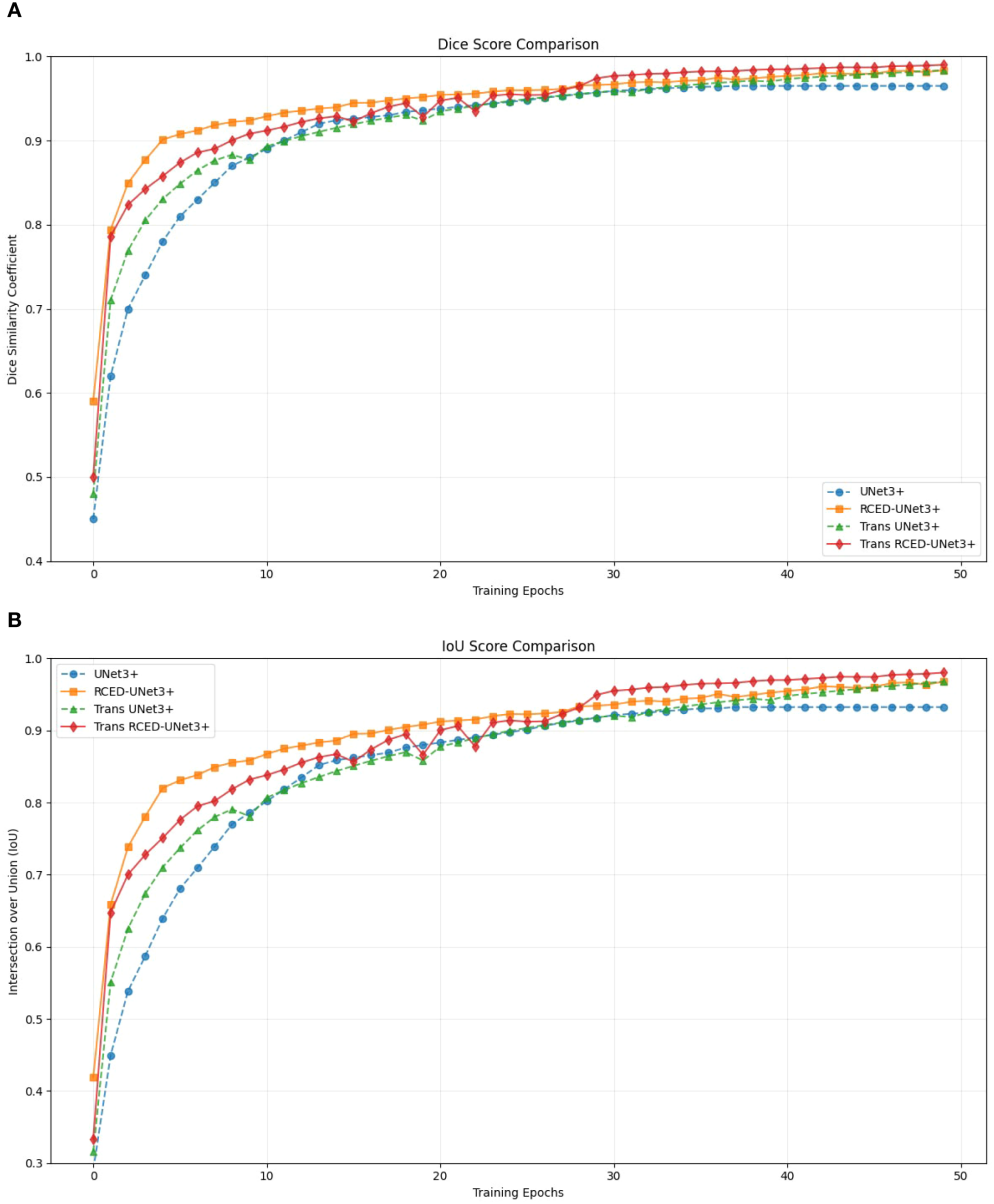
Segmentation performance progression **(A)** Dice score of UNet3+, RCED-UNet3+, Trans UNet3+, and Trans RCED-UNet3+ **(B)** Intersection over Union (IoU) of UNet3+, RCED-UNet3+, Trans UNet3+, and Trans RCED-UNet3+.

**Figure 5** presents the progression of Dice and IoU metrics of UNet3+, RCED-UNet3+, Trans UNet3+, and Trans RCED-UNet3+ across training epochs. The UNet3+ model shows the steady but plateaus at a comparatively lower accuracy. By enhancing the UNet3+ model with residual connections in the encoder and decoder, the RCED-UNet3+ architecture stabilizes more quickly and achieves higher results. The Trans UNet3+ converges more quickly and achieves higher accuracy by incorporating a transformer in the UNet3+ model, which recognizes long-range spatial dependencies and global contextual information. The combination of RCED-UNet3+ and Trans UNet3+ architecture, the Trans RCED-UNet3+ exhibits both faster convergence and greater stability, maintaining a consistent upward trend throughout training.

**Figure 6:**
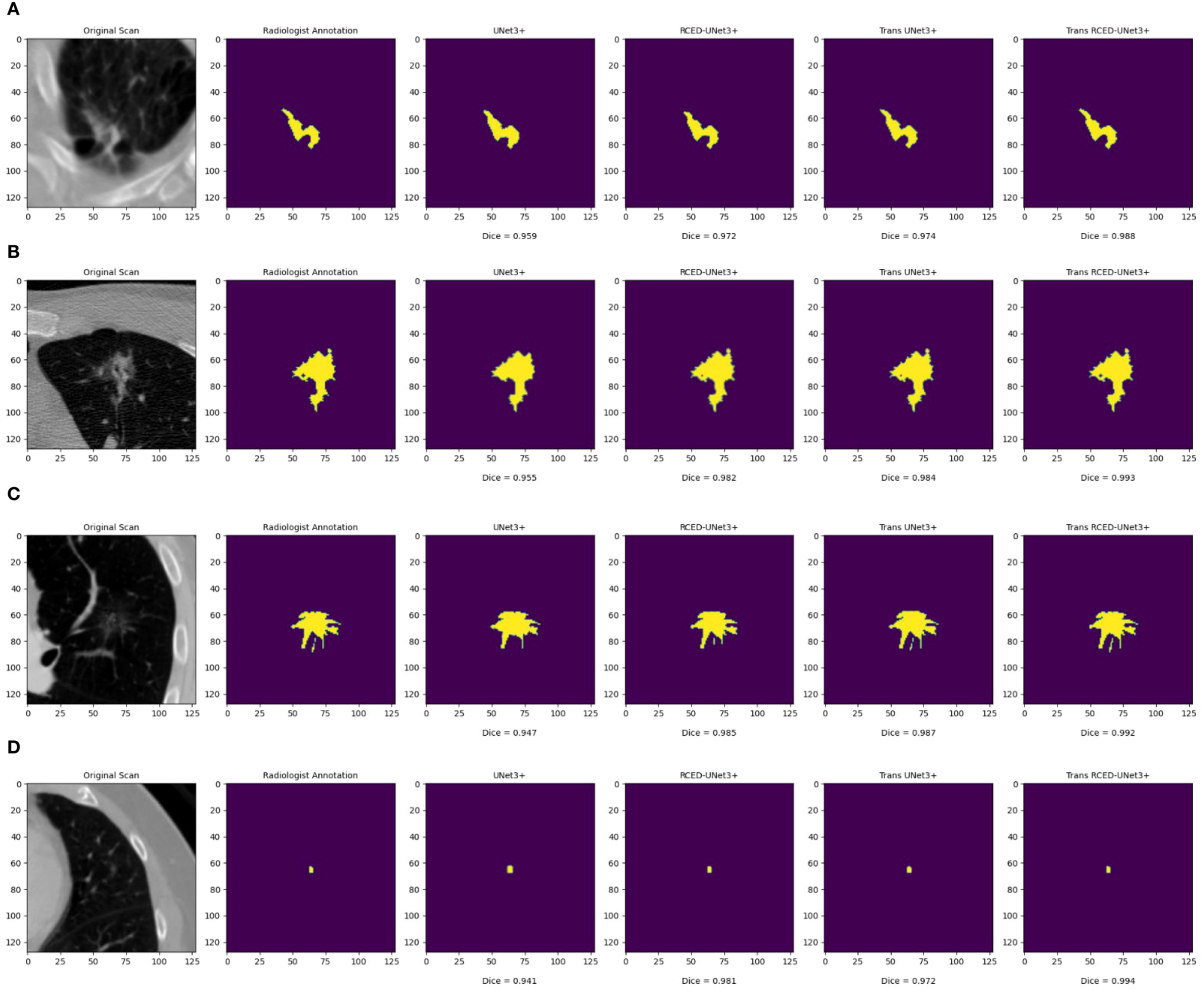
Segmentation results across nodules of varying sizes: **(A)** medium irregular lesion, **(B)** large heterogeneous mass, **(C)** medium spiculated lesion, and **(D)** small homogeneous nodule.

**Figure 6** presents the segmentation results across nodules of various sizes, revealing that Trans RCED- UNet3+ achieves dice score values ranging from 0.988 to 0.994. This shows that the model effectively segments irregular, heterogeneous, spiculated nodules and additionally shows high accuracy for smaller homogeneous cases. This is due to the enhancement of the residual connection in the encoder and decoder, and the bottleneck transformer.

**Figure 7:**
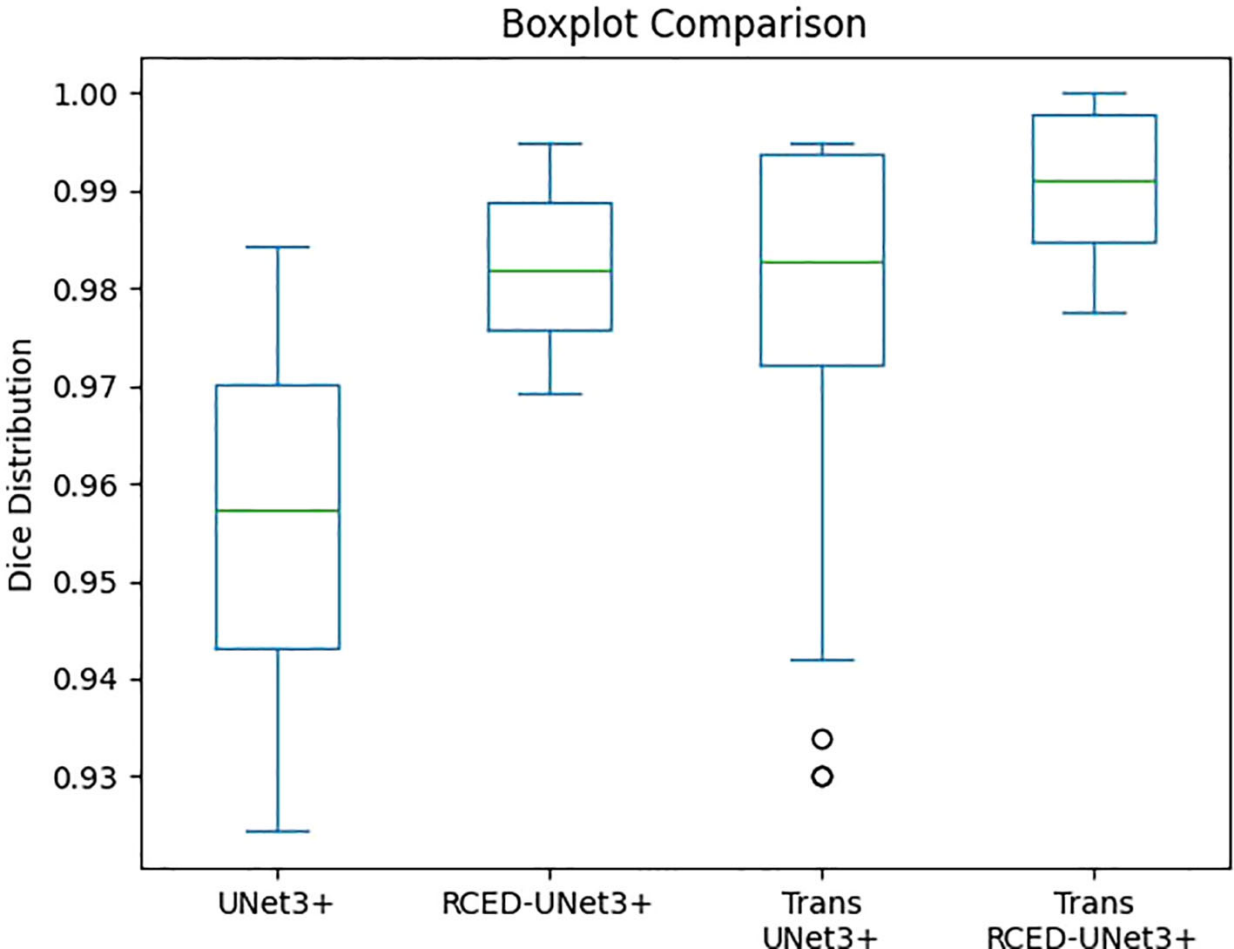
Comparison of the segmentation performance of UNet3+, RCED-UNet3+, Trans UNet3+ and Trans RCED-UNet3+ using the boxplot.

**Figure 7** illustrates the box plot comparison of UNet3+, RCED-UNet3+, Trans UNet3+ and Trans RCED-UNet3+ shows clear performance differences. The findings reveal that UNet3+ exhibited the lowest performance, followed by RCED-UNet3+ and Trans UNet3+ while Trans-RCED-UNet3+ achieved the best results. This demonstrates that incorporating residual connections into UNet3+ and integrating a transformer within the bottleneck of RCED-UNet3+ effectively enhanced the model’s performance.

### 5.3 Loss function and hyperparameter optimization

The hybrid loss function used in RCED-UNet3+ has been retained and proven effective for lung nodule segmentation. However, with the integration of transformer-based self-attention, an attention-weighted loss function could further enhance performance. While the current loss function ensures strong overlap accuracy (Dice = 0.990, IoU = 0.981), an attention-guided approach might:

- Prioritize high-attention regions, improving the segmentation of small and complex lung nodules.
- Adapt dynamically to feature importance, reducing misclassification in challenging cases.
- Better integrate transformer capabilities by reinforcing areas with strong feature responses.

The performance improvements of Trans-RCEDUNet3+ are also partly due to the careful tuning of hyperparameters for the transformer module:

- Fine-tuning the number of attention heads and FFN size significantly improved segmentation accuracy, especially for small lung nodules.
- A dropout rate of 0.15 effectively prevented overfitting, enabling the model to generalize well across the LIDC-IDRI dataset.
- Grid search and random search methods ensured that the model avoided unnecessary computational overhead while maintaining optimal performance.

**Figure 8:**
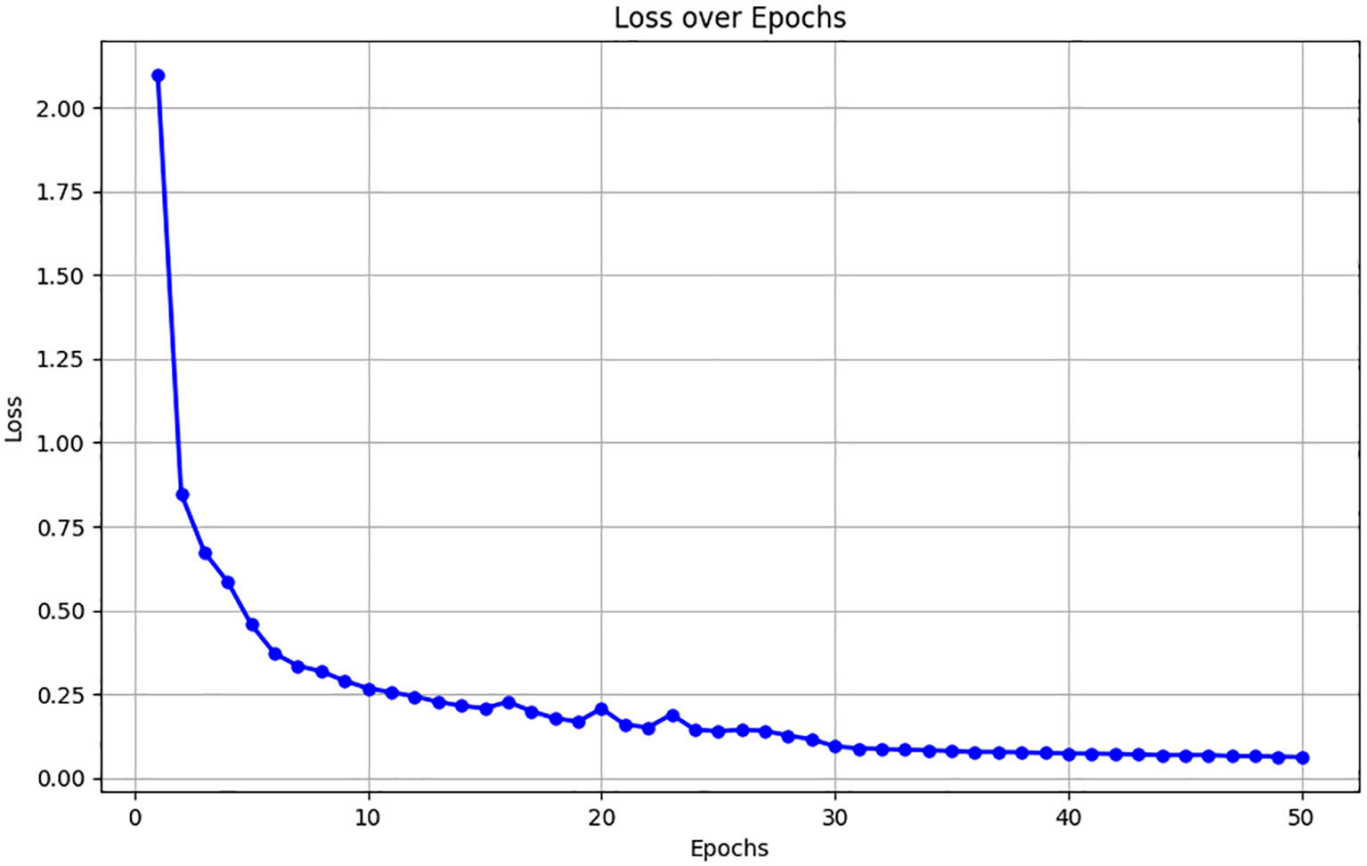
Changes in hybrid loss throughout training epochs on the training dataset.

**Figure 8** presents the changes in the hybrid loss throughout the training epochs, a gradual decrease in the loss value reflects the model’s improved learning efficiency and its increasing effectiveness in tackling the challenges associated with lung nodule segmentation.

### 5.4 Performance evaluation and comparative analysis

The Trans-RCEDUNet3+ model was compared with nine other state-of-the-art lung nodule segmentation models, as shown in **Table 6**. The results highlight the superior performance of Trans-RCEDUNet3+, which achieves a Dice score of 0.990, outperforming all other models.

**Table 6:** Comparison of Trans RCED-UNet architecture with 09 other state-of-the-art lung nodule segmentation architectures.

Model	Dataset(s)	Architecture	Dice score	Preprocessing	Data augmentation	Framework	Optimizer	Learning rate	Batch size	Loss function
SALM (38)	LIDC-IDRI	Vision Transformer + CNN hybrid	0.930	HU normalized (W:1500, L: -160), scaled to [0,255], isotropic resampling to 1×1×1 mm	Random flips, rotations (-20° to +20°), scaling (0.8× to 1.2×)	PyTorch 2.0	Adam	1×10^−4^	8 (2D), 1 (3D)	0.5 × BCE + 0.5 × Dice
MCAT-Net (39)	LIDC-IDRI	Multi-threshold + Coordinate Attention Transformer	0.882	HU clipped [-1100, +450], lung mask applied, cropped around nodules	Not specified	PyTorch	Adam	0.001	128	Not specified
EDT Net (40)	LIDC-IDRI	Edge Detection + Transformer Network	0.842	ROI cropping, resized to 256×256, cancerous nodule regions segmented	Horizontal/vertical flips, 90°/180° rotations, additional cropping	TensorFlow 2.1	Adam	0.0001	64	α × Dice + (1-α) × Cross-Entropy
Trans AttUNet (41)	JSRT and NIH	U-Net + Transformer Attention	0.865	Data set-specific resizing.	Not specified	PyTorch	SGD	0.0001	4	Sorensen-Dice + Binary Cross-Entropy
DMC-UNet (42)	LIDC-IDRI,	Dual Multi-Scale Convolution U-Net	0.899	DICOM to PNG conversion, manual annotation, cropped, and centered on nodules	Horizontal flips, brightness/ contrast/ saturation/ adjustments (FAHGMU only)	PyTorch 1.12.1	Adam (LIDC-IDRI) Adam (FAHGMU)	0.0001 (LIDC-IDRI) 0.001 (FAHGMU)	16 (LIDC-IDRI) 16 (FAHGMU)	Cross-Entropy (FAHGMU), Dice + BCE (LIDC- IDRI)
CTBP-Net (43)	LIDC-IDRI	Cross-Transformer Bidirectional Pyramid	0.916	Cropped patches centered on nodules, nodules smaller than 4mm is removed	Random rotation, Gaussian noise, brightness/contrast adjustments	PyTorch	SGD	0.001	12	BCE + Dice + Hausdorff Distance
HT-Net (44)	LUNA	Hierarchical Transformer Network	0.987	Normalized to [0,1] then [-1,1], resized from original 128 ×128	Flips, rotations	PyTorch	Adam	1×10^−4^	Not specified	Dice coefficient loss
DPBET (45)	LIDC-IDRI	Dual-Path Boundary Enhancement Transformer	0.898	Cropped to 64×64 centered on nodules, nodules >4mm diameter	Rotation, translation, flipping	Not specified	SGD	0.001	8	Not specified
TransUNet (31)	LIDC-IDRI	Hybrid CNN–Transformer UNet	0.871	HU clipped [-1000, +400], normalized, ROI centered on nodules (>3mm), ROI and lung parenchyma Extraction.	Horizontal flip, vertical flip, random 90° rotation, transpose operations.	PyTorch 1.10	Adam	0.00005	8	Not specified
Tran RCED-UNet3+	LIDC-IDRI	Residual Connection in Encoder Decoder + Bottle neck Transformer	0.990	Lung parenchyma is extracted from CT images to create a precise lung mask that supports accurate segmentation	horizontal flipping, vertical flipping, and rotation	PyTorch	SGD	0.001	32	Attention-Weighted Hybrid Loss (Dice + BCE)

**Table 6** presents a comprehensive comparison between the proposed Trans RCED-UNet3+ and nine advanced lung nodule segmentation models. The analysis includes the datasets, architectural structures, preprocessing techniques, augmentation methods, implementation platforms, optimization methods, and loss functions. With a Dice score of 0.990, Trans RCED-UNet3+ outperforms ten leading architectures, showcasing its superior segmentation capability. It exceeds SALM (0.930), which incorporates Vision Transformers and utilizes a refined positional encoding strategy. It also surpasses MCAT (0.882), a model that integrates a Multi-threshold Feature Separation Module for texture enhancement, a Coordinate Attention Mechanism for spatial precision, and Transformers for improved global feature learning. Additionally, it outperforms EDTNet (0.842), which employs successive transformer blocks, patch-expanding layers, and both down-sampling and up-sampling mechanisms to refine segmentation performance. Furthermore, HT-Net (0.987), known for its multi-scale and hierarchical context modules, falls short in comparison. Trans RCED-UNet3+ also delivers superior results over CTBP-Net (0.916), both of which integrate convolution-transformer techniques and cross-transformer mechanisms. It also surpasses DMC-UNet (0.899), which leverages a compact residual framework with multiscale aggregation, and TransAttUnet (0.865), which utilizes self-aware attention inspired by transformers along with multi-scale skip connections. The approach ultimately yields superior segmentation accuracy over DPBET (0.898), a method that utilizes a dual-path architecture in combination with a hybrid transformer. The Trans RCED-UNet3+ model achieves high dice score is due to its transformer-based bottleneck neck, which recognizes long-range spatial dependencies and global contextual information along with a residual connection, which solves the problem of vanishing gradient.

### 5.5 Assessment using additional metrics

**Table 7** presents assessment of the model using Accuracy, Precision, and Recall, which prvides more detailed evaluation of the model’s segmentation effectiveness.

**Table 7:** Evaluation of accuracy, precision, and recall for RCED-UNet3+ and Trans-RCED-UNet3+ models trained on augmented datasets.

Model	Accuracy	Precision	Recall
RCED-UNet3+	0.99	0.98	0.97
Trans RCED-UNet3+	0.99	0.98	0.99

## 6 Conclusion

The Trans RCED-UNet3+ architecture achieves high segmentation accuracy compared to the RCED-UNet3+ by incorporating a transformer-based bottleneck, which recognizes long-range spatial dependencies and global contextual information across the image. The model achieved a Dice score of 0.990, surpassing the accuracy of 0.984. These results are attributed to the combination of convolutional structures with transformer components to address the challenges of medical image segmentation, particularly to accurately detect nodules, including those attached to the lung walls. Future research will explore the application of Trans RCED-UNet3+ across a variety of medical imaging datasets to assess its robustness under different conditions. Enhancing transformer modules and attention mechanisms is also expected to further improve accuracy and computational efficiency. The findings demonstrate promising potential for advancing automated medical image analysis toward greater accuracy and reliability.

## Data Availability

The original contributions presented in the study are included in the article/supplementary material. Further inquiries can be directed to the corresponding authors.
